# Using high-resolution variant frequencies to empower clinical genome interpretation

**DOI:** 10.1038/gim.2017.26

**Published:** 2017-05-18

**Authors:** Nicola Whiffin, Eric Minikel, Roddy Walsh, Anne H O’Donnell-Luria, Konrad Karczewski, Alexander Y Ing, Paul J R Barton, Birgit Funke, Stuart A Cook, Daniel MacArthur, James S Ware

**Affiliations:** 1 Cardiovascular Genetics and Genomics, National Heart and Lung Institute, Imperial College London, London, UK; 2 NIHR Royal Brompton Cardiovascular Biomedical Research Unit, Royal Brompton & Harefield Hospitals & Imperial College London, London, UK; 3 Analytic & Translational Genetics Unit, Massachusetts General Hospital, Boston, Massachusetts, USA; 4 Program in Medical and Population Genetics, Broad Institute of MIT & Harvard, Cambridge, Massachusetts, USA; 5 Laboratory for Molecular Medicine, Partners HealthCare Personalized Medicine, Cambridge, Massachusetts, USA; 6 Department of Pathology, Massachusetts General Hospital and Harvard Medical School, Boston, Massachusetts, USA; 7 National Heart Centre Singapore, Singapore, Singapore; 8 Duke-National University of Singapore, Singapore, Singapore; 9 Department of Medicine, Harvard Medical School, Boston, Massachusetts, USA; 10 MRC London Institute of Medical Sciences, Imperial College London, London, UK

**Keywords:** allele frequency, clinical genomics, ExAC, inherited cardiovascular conditions, variant interpretation

## Abstract

**Purpose:**

Whole-exome and whole-genome sequencing have transformed the discovery of genetic variants that cause human Mendelian disease, but discriminating pathogenic from benign variants remains a daunting challenge. Rarity is recognized as a necessary, although not sufficient, criterion for pathogenicity, but frequency cutoffs used in Mendelian analysis are often arbitrary and overly lenient. Recent very large reference datasets, such as the Exome Aggregation Consortium (ExAC), provide an unprecedented opportunity to obtain robust frequency estimates even for very rare variants.

**Methods:**

We present a statistical framework for the frequency-based filtering of candidate disease-causing variants, accounting for disease prevalence, genetic and allelic heterogeneity, inheritance mode, penetrance, and sampling variance in reference datasets.

**Results:**

Using the example of cardiomyopathy, we show that our approach reduces by two-thirds the number of candidate variants under consideration in the average exome, without removing true pathogenic variants (false-positive rate<0.001).

**Conclusion:**

We outline a statistically robust framework for assessing whether a variant is “too common” to be causative for a Mendelian disorder of interest. We present precomputed allele frequency cutoffs for all variants in the ExAC dataset.

## Introduction

Whole-exome and whole-genome sequencing have been instrumental in identifying causal variants in Mendelian disease patients.^1^ As every individual harbors ~12,000–14,000 predicted protein-altering variants,^2^ distinguishing disease-causing variants from benign bystanders is perhaps the principal challenge in contemporary clinical genetics. A variant’s low frequency in, or absence from, reference databases is recognized as a necessary, but not sufficient, criterion for variant pathogenicity.^3, 4^ The recent availability of very large reference databases, such as the Exome Aggregation Consortium (ExAC)^2^ dataset, which has characterized the population allele frequencies (AFs) of 10 million genomic variants through analysis of exome sequencing data from over 60,000 humans, provides an opportunity to obtain robust frequency estimates even for rare variants, improving the theoretical power for AF filtering in Mendelian variant discovery efforts.

In practice, there exists considerable ambiguity around what AF should be considered “too common,” with the lenient values of 1 and 0.1% often invoked as conservative frequency cutoffs for recessive and dominant diseases respectively.^5^ Population genetics, however, dictates that severe disease-causing variants must be much rarer than these cutoffs, except in cases of bottlenecked populations, balancing selection, or other special circumstances.^6, 7^

It is intuitively apparent that when assessing a variant for a causative role in a dominant Mendelian disease, the frequency of a variant in a reference sample, not selected for the condition, should not exceed the prevalence of the condition.^8, 9^ This rule must, however, be refined to account for different inheritance modes, genetic and allelic heterogeneity, and reduced penetrance. In addition, for rare variants, estimation of true population AF is clouded by considerable sampling variance, even in the largest samples currently available. These limitations have encouraged the adoption of very lenient AF filtering approaches,^10, 11^ and recognition that more stringent approaches that account for disease-specific genetic architecture are urgently needed.^8^

Here we present a statistical framework for assessing whether variants are sufficiently rare to cause penetrant Mendelian disease, while accounting for both architecture and sampling variance in observed allele counts (ACs). We demonstrate that AF cutoffs well below 0.1% are justified for a variety of human disease phenotypes and that such filters can remove an additional two-thirds of variants from consideration compared to traditionally lenient frequency cutoffs, without discarding true pathogenic variants. We present precomputed AF filtering values for all variants in the ExAC database, for comparison with user-defined disease-specific thresholds, which are available through the ExAC data browser and for download, to assist others in applying this framework.

## Materials and methods

### Defining the statistical framework

We define a two-stage approach to determine whether a variant observed in a reference sample is too common to cause a given disease. First, we define a maximum population AF that we believe is credible for a pathogenic variant, given the genetic architecture of the disease in question. Second, we determine whether the observed allele count in our reference sample is consistent with a variant having this frequency in the population from which the sample was drawn.

For a penetrant dominant Mendelian allele to be disease-causing, it cannot be present in the general population more frequently than the disease it causes. Furthermore, if the disease is genetically heterogeneous, it must not be more frequent than the proportion of cases attributable to that gene, or indeed to any single variant. We can therefore define the maximum credible population AF (for a pathogenic allele) as:


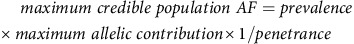


where *maximum allelic contribution* is the maximum proportion of cases potentially attributable to a single allele, a measure of heterogeneity.

For recessive conditions, the maximum AF is defined as:


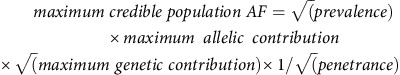


where *maximum genetic contribution* represents the proportion of all cases that are attributable to the gene under evaluation, and *maximum allelic contribution* represents the proportion of cases attributable to that gene that are attributable to an individual variant (see Supplementary Methods for full derivation).

Disease prevalence estimates were obtained from the literature and taken as the highest value reported. Cardiovascular disease variants were modeled with a penetrance of 0.5, corresponding to the reported penetrance of the hypertrophic cardiomyopathy (HCM) variant used to illustrate our approach^12^ and the minimum found across a range of variants/disorders.

We do not know the true population AF of any variant, having only an observed AF in a finite population sample. Moreover, confidence intervals around this observed frequency are problematic to estimate given our incomplete knowledge of the frequency spectrum of rare variants, which is skewed toward very rare variants. For instance, a variant observed only once in a sample of 10,000 chromosomes is much more likely to have a frequency<1:10,000 than a frequency >1:10,000.^2^

To address this, we begin by specifying a maximum *true* AF value we are willing to consider in the population (using the equation above), from which we can estimate the probability distribution for allele counts in a given sample size (see Supplementary Methods online). This allows us to set an upper limit on the number of alleles in a sample that is consistent with a given underlying population frequency. For example, a variant with a true population AF of 0.0001 would be expected to occur in a sample of 100,000 alleles ≤15 times with a probability of 0.95.

We therefore computed a maximum tolerated allele count (AC) as the AC at the upper bound of the one-tailed 95% confidence interval (95%CI AC) of a Poisson distribution, for the specified maximum credible AF, given the sample size (observed allele number, AN).

### Precomputing filtering AF values for ExAC

We can reverse this process to determine the maximum true population AF that is consistent with a particular observed sample AC, and we applied this to the ExAC dataset (version 0.3.1). In order to precompute filtering AF values for all variants in ExAC, we apply a two-step approach to the AC and AN values for each of the five major continental populations, and take the highest result from any population (more explanation in Supplementary Methods).

1. We use R’s uniroot function to find an AF value (though not necessarily the highest AF value) for which the 95%CI AC is one less than the observed AC.
2. We loop, incrementing by units of millionths, and return the highest AF value that still gives a 95%CI AC less than the observed AC.

We used adjusted AC and AN, meaning variant calls with GQ (genotype quality)≥20 and DP (depth of coverage)≥10.

### Simulated Mendelian variant discovery analysis

To simulate Mendelian variant discovery, we randomly selected 100 individuals from each of five major continental populations and filtered their exomes against filtering AFs derived from the remaining 60,206 ExAC individuals. The subset of individuals was the same as that previously reported.^2^ Predicted protein-altering variants are defined as missense and equivalent (including in-frame indels, start lost, stop lost, and mature miRNA-altering), and protein-truncating variants (nonsense, essential splice site, and frameshift).

### Variant curation

Pathogenic and nonconflicted variants were extracted from ClinVar (9 July 2015 release) as described previously.^2^ ExAC counts were determined by matching on chromosome, position, reference, and alternate alleles. For all variants above the proposed maximum tolerated AC for HCM, literature from both the Human Gene Mutation Database and PubMed was reviewed and the level of evidence supporting pathogenicity was curated according to the criteria of the American College for Medical Genetics and Genomics (ACMG).^3^

### Calculating odds ratios for HCM variant burden

A total of 322 HCM patients and 852 healthy volunteers (both confirmed by cardiac MRI) recruited to the NIHR Royal Brompton cardiovascular BRU were sequenced using the IlluminaTruSight Cardio Sequencing Kit^13^ on Illumina MiSeq and NextSeq platforms. This study had ethical approval (REC: 09/H0504/104+5) and informed consent was obtained for all subjects. The number of rare variants in the eight sarcomeric genes associated with HCM (*MYBPC3*, *MYH7*, *TNNT2*, *TNNI3*, *MYL2*, *MYL3*, *TPM1* and *ACTC1*) were calculated for all protein-altering variants (frameshift, nonsense, splice donor/acceptor, missense and in-frame insertions/deletions), with case/control odds ratios calculated separately for nonoverlapping ExAC AF bins with the following breakpoints: 4 × 10^−5^, 1 × 10^−4^, 5 × 10^−4^, and 1 × 10^−3^. Odds ratios were calculated as OR=(cases with variant/cases without variant)/(controls with variant/controls without variant).

## Results

### Application and validation in hypertrophic cardiomyopathy

#### Defining maximum credible population AF

We illustrate our generalizable approach using the dominant cardiac disorder HCM, which has an estimated prevalence of 1 in 500 in the general population.^14^ As there have been previous large-scale genetic studies of HCM, with series of up to 6,179 individuals,^14, 15^ we can assume that no newly identified variant will be more common in cases than those identified to date (at least for well-studied ancestries), allowing us to define the maximum contribution of any single variant to the disorder. In these series, the largest proportion of cases is attributable to the missense variant *MYBPC3* c.1504C>T (p.Arg502Trp), found in 104 of 6,179 HCM cases (1.7% 95%CI 1.4–2.0%).^14, 15^ We therefore take the upper bound of this proportion (0.02) as an estimate of the maximum allelic contribution in HCM (Table 1). Our maximum expected population AF for this allele, assuming penetrance 0.5 as previously reported,^12^ is 1/500 × 1/2 (dividing prevalence per individual by the number of chromosomes per individual) × 0.02 × 1/0.5=4.0 × 10^−5^, which we take as the maximum credible population AF for any causative variant for HCM (Table 1).

#### Controlling for sample variation

To apply this threshold while remaining robust to chance variation in observed ACs, we ask how many times a variant with population AF of 4.0 × 10^−5^ can be observed in a random population sample (see “Materials and Methods”). At a 5% error rate, this yields a maximum tolerated AC of 9, assuming 50% penetrance (5 for fully penetrant alleles) for variants genotyped in the full ExAC cohort (sample size=121,412 chromosomes). The *MYBPC3*:c.1504C>T variant is observed 3 times in ExAC (freq=2.49 × 10^−5^; Table 1).

To facilitate these calculations, we have developed an online calculator (http://cardiodb.org/alleleFrequencyApp) that will compute maximum credible population AF and maximum sample AC for a user-specified genetic architecture, and conversely allow users to dynamically explore what genetic architecture(s) might be most compatible with an observed variant having a causal role in disease.

#### Assessing the accuracy of our approach

For all diseases with case series that permitted us to define the genetic architecture, the commonest variant in the case series was well within the calculated maximum AC in ExAC (Table 1).

To assess the HCM thresholds empirically, we explored the ExAC AF spectrum of 1,132 distinct autosomal variants, identified in 6,179 published HCM cases referred for diagnostic sequencing, and individually assessed and clinically reported according to international guidelines.^14, 15^ 477/479 (99.6%) of variants reported as “pathogenic” or “likely pathogenic” fell below our threshold (Figure 1), including all variants with a clear excess in cases. The 2 variants historically classified as “likely pathogenic,” but prevalent in ExAC in this analysis, were reassessed using contemporary ACMG criteria: there was no strong evidence in support of pathogenicity, and they were reclassified in light of these findings (Supplementary Table S1). This analysis identifies 66/653 (10.1%) variants of unknown significance (VUS) that are very unlikely to be causative for HCM.

The above analysis applied a single global AC limit of 9 for HCM; however, as AFs differ between populations, filtering based on frequencies in individual populations may provide greater power.^2^ For example, a variant relatively common in any one population is unlikely to be pathogenic, even if rare in other populations, provided the disease prevalence and architecture are consistent across populations. We therefore compute a maximum tolerated AC for each distinct subpopulation of our reference sample, and filter based on the highest AF observed in any major continental population (see “Materials and Methods”). The tightness of the Poisson distribution used to compute maximum tolerated AC is a function of sample size, and so our approach is more conservative when the AN is lower, thus avoiding inappropriately filtering variants based on chance observation of a few alleles in a smaller subpopulation or at a poorly genotyped site (see Supplementary Note 3).

To further validate this approach, we examined all 601 variants identified in ClinVar^16^ as “pathogenic” or “likely pathogenic” and nonconflicted for HCM. Of these, 558 (93%) were sufficiently rare when assessed as described. 43 variants were insufficiently rare in at least one ExAC population, and were therefore recurated. 42 of these had no segregation or functional data sufficient to demonstrate pathogenicity in the heterozygous state, and would be classified by the contemporary ACMG framework as VUS at most. The remaining variant (*MYBPC3*:c.3330+5G>C) had convincing evidence of pathogenicity, though with uncertain penetrance (see Supplementary Methods), and was observed twice in the African/African-American ExAC population. This fell outside the 95% confidence interval for an underlying population frequency <4 × 10^−5^, but within the 99% confidence threshold: a single outlier due to stochastic variation is unsurprising given that these nominal probabilities are not corrected for multiple testing across 601 variants. In light of our updated assessment, 20 variants were reclassified as benign/likely benign and 22 as VUS, according to the ACMG guidelines for variant interpretation^3^ (Supplementary Table S1).

After curating variants above our calculated HCM threshold, the false-positive rate was 0/477 (0.000; 95%CI 0.000–0.008) and 1/559 (0.002; 95%CI 0.000–0.010) for the published HCM cohort and ClinVar data respectively.

#### Extending this approach to other disorders

This framework relies on estimation of the genetic architecture of a condition, which may not be well described. For diseases where large case series are absent, we can estimate the genetic architecture parameters by extrapolating from similar disorders and/or variant databases.

Where disease-specific variant databases exist, we can use these to estimate the maximum allelic contribution. For example, Marfan syndrome is a rare connective-tissue disorder caused by variants in the *FBN1* gene. The UMD-FBN1 database^17^ contains 3,077 variants in *FBN1* from 280 references (last updated 28 August 2014). The most common variant is in 30/3,006 records (1.00% 95% CI 0.53–1.46%), which likely overestimates its contribution to disease if related individuals are not systematically excluded. Taking the upper bound of this frequency as our maximum allelic contribution, we derive a maximum tolerated AC of 2 (Table 2). None of the five most common variants in the database are present in ExAC.

Where no mutation database exists, we can use what is known about similar disorders to estimate the maximum allelic contribution. For the better-characterized cardiac conditions in Table 1, the maximum proportion of cases attributable to any one variant is 6.7% (95% CI 4.1–9.2% *PKP2*:c.2,146-1G>C found in 24/361 ARVC cases^15^). We therefore propose the upper bound of this confidence interval (rounded up to 0.1) as a reasonable estimate of the maximum allelic contribution for other genetically heterogeneous cardiac conditions, unless there is disease-specific evidence to alter it. For Noonan syndrome and Catecholaminergic Polymorphic Ventricular Tachycardia (CPVT-an inherited cardiac arrhythmia syndrome) with prevalences of 1 in 1,000^18^ and 1 in 10,000^19^ respectively, this translates to maximum population frequencies of 5 × 10^−5^ and 5 × 10^−6^ and maximum tolerated ExAC ACs of 10 and 2 (Table 2).

Finally, if the allelic heterogeneity of a disorder is not well characterized, it is conservative to assume minimal heterogeneity, so that the contribution of each gene is modeled as attributable to one allele, and the maximum allelic contribution is substituted by the maximum genetic contribution (i.e., the maximum proportion of the disease attributable to a single gene). For classic Ehlers-Danlos syndrome, up to 40% of the disease is caused by variation in the *COL5A1* gene.^20^ Taking 0.4 as our maximum allelic contribution, and a population prevalence of 1/20,000,^20^ we derive a maximum tolerated ExAC AC of 5 (Table 2).

Here we have illustrated frequencies analyzed at the level of the disease. In some cases this may be further refined by calculating distinct thresholds for individual genes, or even variants. For example, if there is one common founder mutation but no other variants that are recurrent across cases, then it would make sense to have the founder mutation as an exception to the calculated threshold.

#### Application to recessive diseases

So far we have considered diseases with a dominant inheritance model. Our framework is readily modified for application in recessive disease, and to illustrate this we consider the example of primary ciliary dyskinesia (PCD), which has a prevalence of up to 1 in 10,000 individuals in the general population.^21^

Intuitively, if one penetrant recessive variant were to be responsible for all PCD cases, it could have a maximum population frequency of 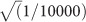. We can refine our evaluation of PCD by estimating the maximum genetic and allelic contribution (see “Materials and Methods”). Across previously published cohorts of PCD cases,^22, 23, 24^ *DNAI1* IVS1+2_3insT was the most common variant with a total of 17/358 alleles (4.7% 95% CI 2.5–7.0%). Given that ~9% of all patients with PCD have disease-causing variants in *DNAI1* and the IVS1+2_3insT variant is estimated to account for ~57% of variant alleles in *DNAI1*,^22^ we can take these values as estimates of the maximum genetic and allelic contribution for PCD, yielding a maximum expected population AF of 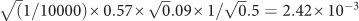. This translates to a maximum tolerated ExAC AC of 322. *DNAI1* IVS1+2_3insT is itself present at 56/121,108 ExAC alleles (45/66,636 non-Finnish European alleles). A single variant reported to cause PCD in ClinVar occurs in ExAC with AC>332 (*NME8* NM_016616.4:c.271-27C>T; AC=2,306/120,984): our model therefore indicates that this variant frequency is too common to be disease-causing, and consistent with this we note that it meets none of the current ACMG criteria for assertions of pathogenicity, and would reclassify it as VUS (see Supplementary Methods).

#### Precomputing threshold values for the ExAC populations

For each ExAC variant, we defined a “filtering AF” that represents the threshold disease-specific “maximum credible AF” at or below which the disease could not plausibly be caused by that variant. A variant with a filtering AF ≥ the maximum credible AF for the disease under consideration should be filtered, while a variant with a filtering AF below the maximum credible remains a candidate. This filtering AF is not disease-specific: it can be applied to any disease of interest by comparing with a user-defined disease-specific maximum credible AF (Figure 2). This value has been precomputed for all variants in ExAC (see “Materials and Methods” and Supplementary Methods), and is available via the ExAC VCF and browser (http://exac.broadinstitute.org).

To assess the efficiency of our approach, we calculated the filtering AF on 60,206 exomes from ExAC and applied these filters to a simulated dominant Mendelian variant discovery analysis on the remaining 500 exomes (see “Materials and Methods”). Filtering at AFs lower than 0.1% substantially reduces the number of predicted protein-altering variants in consideration, with the mean number of variants per exome falling from 176 to 63 at cutoffs of 0.1 and 0.0001% respectively (Figure 3a). Additionally, we compared the prevalence of variants in HCM genes in cases and controls across the AF spectrum, and computed disease odds ratios for different frequency bins. The odds ratio for disease-association increases markedly at very low AFs (Figure 3b), demonstrating that increasing the stringency of a frequency filter improves the information content of a genetic result.

## Discussion

We have outlined a statistically robust framework for assessing whether a variant is “too common” to be causative for a Mendelian disorder of interest. To our knowledge, there is currently no equivalent guidance on the use of variant frequency information, resulting in inconsistent thresholds across both clinical and research settings. Furthermore, though disease-specific thresholds are recommended,^8^ in practice the same thresholds may be used across all diseases, even where they have widely differing genetic architectures and prevalences. We have shown the importance of applying stringent AF thresholds, in that many more variants can be removed from consideration, and the remaining variants have a much higher likelihood of being relevant. We also show, using HCM as an example, how lowering this threshold does not remove true dominant pathogenic variants.

To assist others in applying our framework, we have precomputed a “filtering AF” for all variants across the ExAC dataset. This is defined such that if the filtering AF of a variant is at or above the user-defined “maximum credible population AF” for the disease in question, then that variant is not a credible candidate (in other words, for any population AF below the threshold value, the probability of the observed AC in the ExAC sample is <0.05). Once a user has determined their “maximum credible population AF,” they may remove from consideration ExAC variants for which the filtering AF is greater than or equal to than the chosen value.

Our method is designed to be complementary to and used alongside other gene and variant level methods to filter and prioritize candidate variants (e.g., gene level constraint,^25^ amino acid conservation^26, 27^ and missense prediction algorithms^28, 29^) along with segregation and functional data.

We recognize several limitations in our approach. First, we are limited by our understanding of the prevalence and genetic architecture of the disease in question: this characterization will vary for different diseases and in different populations, though we illustrate approaches for estimation and extrapolation of parameters. In particular, we must be wary of extrapolating to or from less well-characterized populations that could harbor population-specific founder mutations. While incomplete knowledge of the genetic architecture of a disease of interest will limit this or any approach to evaluating a specific variant that has been observed at low frequency in a reference population, our framework and accompanying web tool do at least transparently define the range of disease architectures that are compatible with the observed data. For example, many neurological disorders have Mendelian forms as well as idiopathic forms with genetic risk factors of modest effect sizes, high allelic and genetic heterogeneity, and/or dramatic variability in the penetrance of different variants.^9, 30, 31, 32^ Reference population AF information alone can never definitively show that a variant possesses no association with disease, but it can still provide sensible constraints. The calculations described here can be used to show that a variant could be causal only if the prevalence of the disease is higher than published estimates, or its penetrance is below a specified value.^33^

Secondly, it is often difficult to obtain accurate penetrance information for reported variants, and it is also difficult to know what degree of penetrance to expect or assume for newly discovered pathogenic variants. Although we would argue that variants with low penetrance have questionable diagnostic utility, our calculator app allows a user to define a range of compatible penetrance for a given AF (see Supplementary Methods), and implements methods to estimate variant penetrance from prevalence data in case and control cohorts as previously described.^9^

Third, while we believe that ExAC is depleted of severe childhood inherited conditions, and not enriched for cardiomyopathies, it could be enriched relative to the general population for some conditions, including Mendelian forms of common diseases such as diabetes or coronary disease that have been studied in contributing cohorts. Where this is possible, the maximum credible population AF should be derived based on the estimated disease prevalence in the ExAC cohort, rather than the population prevalence.

Finally, although the resulting AF thresholds are more stringent than those previously used, they are likely to still be very lenient for many applications. For instance, we base our calculation on the most prevalent known pathogenic variant from a disease cohort. For HCM, for which more than 6,000 people have been sequenced, it is unlikely that any single newly identified variant, not previously cataloged in this large cohort, will explain a similarly large proportion of the disease as the most common causal variant, at least in well-studied populations. Future work may therefore involve modeling the frequency distribution of all known variants for a disorder, to further refine these thresholds.

The power of our approach is limited by currently available datasets. Increases in both the ancestral diversity and the size of reference datasets will bring additional power to our method over time. We have avoided filtering on variants observed only once, because a single observation provides little information about true AF (see Supplementary Methods). A 10-fold increase in sample size, resulting from projects such as the US Precision Medicine Initiative, will separate vanishingly rare variants from those whose frequency really is ~1 in 100,000. Increased phenotypic information linked to reference datasets will also reduce limitations due to uncertain disease status, and improve prevalence estimates, adding further power to our approach.

### Data and code availability

All data and code required to reproduce the analysis, figures, and manuscript (compiled in R) are available at https://github.com/ImperialCardioGenetics/frequencyFilter. Curated variant interpretations are deposited in ClinVar under the submission name “HCM_ExAC_frequency_review_2016.” ExAC annotations are available at http://exac.broadinstitute.org. Our AF calculator app is located at http://cardiodb.org/alleleFrequencyApp, with source code available at http://github.com/jamesware/alleleFrequencyApp.

## Figures and Tables

**Figure 1:**
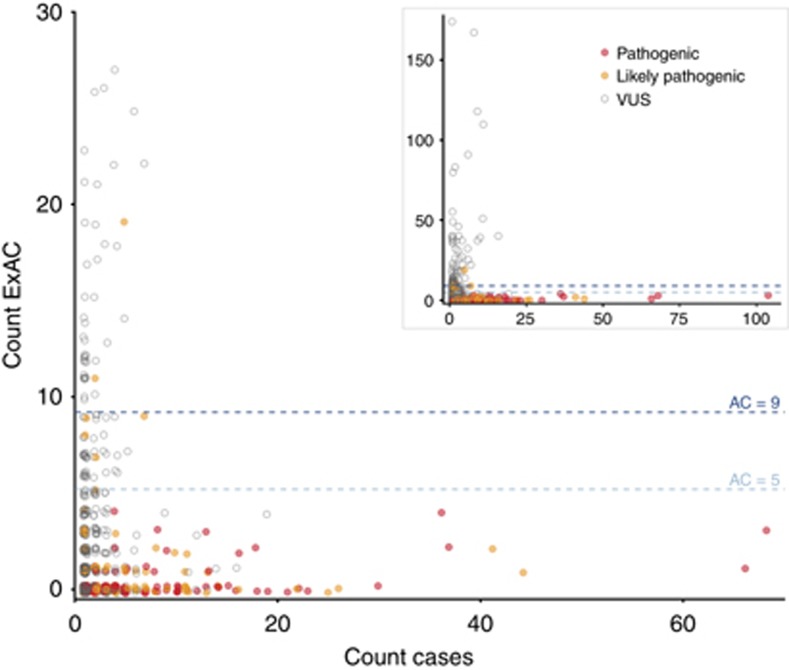
**Plot of Exome Aggregation Consortium (ExAC) allele count (all populations) against case allele count for variants classified as variants of unknown significance (VUS), likely pathogenic, or pathogenic in 6,179 cases of hypertrophic cardiomyopathy.** The dotted lines represent the maximum tolerated ExAC allele counts in hypertrophic cardiomyopathy for 50% (dark blue) and 100% (light blue) penetrance. Variants are color-coded according to reported pathogenicity. Where classifications from contributing laboratories were discordant, the more conservative classification is plotted. The inset panel shows the full dataset; the main panel expands the region of primary interest. True pathogenic variants appropriately fall below our derived allele count threshold.

**Figure 2:**
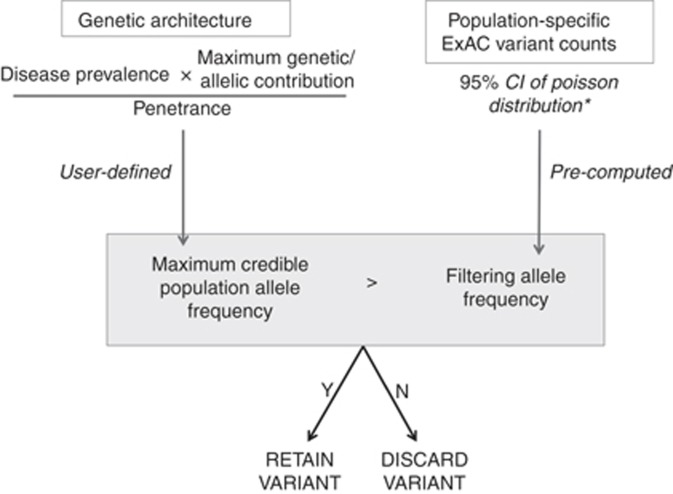
**A flow diagram of our approach, applied to a dominant condition, and using Exome Aggregation Consortium (ExAC) as our reference sample.** First, a disease-level maximum credible population allele frequency (AF) is calculated, based on disease prevalence, heterogeneity, and penetrance. To evaluate a specific variant, we determine whether the observed variant allele count is compatible with disease by comparing this maximum credible population AF against the (precalculated) filtering AF for the variant. *While filtering AF has been precomputed for ExAC variants, the same framework can be readily applied using another reference sample.

**Figure 3:**
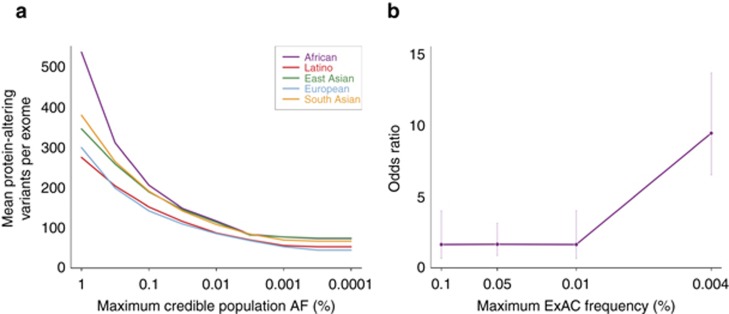
**The clinical utility of stringent allele frequency (AF) thresholds.** (**a**) The number of predicted protein-altering variants (definition in “Materials and Methods”) per exome as a function of the AF filter applied. A one-tailed 95% confidence interval is used, meaning that variants were removed from consideration if their AC would fall within the top 5% of the Poisson probability distribution for the user’s maximum credible AF (x axis). (**b**) The odds ratio for HCM disease-association against AF. The disease odds ratio of a burden test for variants in HCM genes is shown, stratified by variant allele frequency. For each AF bin, the prevalence of variants in sarcomeric HCM-associated genes (*MYH7*, *MYBPC3*, *TNNT2*, *TNNI3*, *MYL2*, *MYL3*, *TPM1*, and *ACTC1*, analyzed collectively) in 322 HCM cases and 852 healthy controls was compared, and an odds ratio computed (see “Materials and Methods”). Data for each bin is plotted at the upper AF cutoff. Error bars represent 95% confidence intervals. The probability that a variant is pathogenic is much greater at very low AFs.

**Table 1:** Details of the most prevalent pathogenic variants in case cohorts for five cardiac conditions

**Disease**	**Prevalence**	**Commonest causative variant**	**Case count**	**Case frequency (95% CI)**	**Penetrance**^a^	**Expected population frequency**	**Model predicted maximum ExAC AC**	**Observed ExAC AC**
HCM	1/500	*MYBPC3*:c.1504C>T	104/6,179	1.7% (1.4–2.0%)	0.5	3.4 × 10^−5^(2.7–4.0 × 10^−5^)	9	3
DCM	1/250	*TNNT2*:c.629_631delAGA	18/1,254	1.4% (0.78–2.1%)	0.5	5.6 × 10^−5^(3.1–8.4 × 10^−5^)	16	0
ARVC	1/1,000	*PKP2*:c.2146-1G>C	24/361	6.7% (4.1–9.2%)	0.5	6.7 × 10^−5^(4.1–9.2 × 10^−5^)	17	6
LQTS	1/2,000	*KCNQ1*:c.797T>C	30/2,500	1.2% (0.77–1.6%)	0.5	6.0 × 10^−6^(3.9–8.2 × 10^−6^)	3	0
Brugada	1/1,000	*SCN5A*:c.5350G>A	14/2,111	0.66% (0.32–1.0%)	0.5	6.6 × 10^−6^(0.32–1.0 × 10^−5^)	3	0

AC, allele count; ARVC, arrhythmogenic right ventricular cardiomyopathy; DCM, dilated cardiomyopathy; ExAC, Exome Aggregation Consortium database; HCM, hypertrophic cardiomyopathy; LQTS, long QT syndrome.

Shown along with the frequency in cases is the estimated population allele frequency (calculated as: case frequency × disease prevalence × 1/2 × 1/variant penetrance) and the observed frequency in the ExAC dataset.

a As penetrance estimates for individual variants are not widely available, we have applied an estimate of 0.5 across these cardiac disorders (see Supplementary Information). Case cohorts and prevalence estimates (taken as the highest value reported) were obtained from HCM,^14, 15^ DCM,^14, 15, 34^ ARVC,^15, 35^ LQTS,^36, 37^ and Brugada.^38, 39^

**Table 2:** Maximum credible population frequencies and maximum tolerated ExAC allele counts for variants causative of exemplar inherited cardiac conditions, assuming a penetrance of 0.5 throughout

**Disease**	**Maximum allelic contribution**	**Prevalence**	**Penetrance**	**Maximum population frequency**	**Maximum tolerated ExAC allele count**
Marfan	0.015	1/3,000	0.5	5.0 × 10^−6^	2
Noonan	0.10	1/1,000	0.5	1.0 × 10^−4^	18
CPVT	0.10	1/10,000	0.5	1.0 × 10^−5^	3
Classic Ehlers-Danlos	0.40	1/20,000	0.5	2.0 × 10^−5^	5

CPVT,catecholaminergic polymorphic ventricular tachycardia; ExAC, Exome Aggregation Consortium database.

Prevalence estimates (taken as the highest value reported) were obtained from Marfan,^40^ Noonan,^18^ CPVT,^19^ and classical Ehlers-Danlos.^20^
