# Supplementary material for: Using high-resolution variant frequencies to empower clinical genome interpretation
Source: Genet Med. 2017 May 18;19(10):1151–8. doi: 10.1038/gim.2017.26 (PMC5563454; doi:10.1038/gim.2017.26)
Supplement: Supplementary file 1 [file gim201726x1.pdf]

Supplementary Material - Using high-resolution variant frequencies to empower clinical genome interpretation

SUPLEMENTARY METHODS

The statistical model

For dominant disorders we define the maximum credible population AF (for a pathogenic allele) as:

maximum credible population AF = prevalence × *maximum allelic contribution* × *1/penetrance*

where *maximum allelic contribution* is the maximum proportion of cases potentially attributable to a single allele, a measure of heterogeneity. It is important to note that although we estimate the *maximum allelic contribution* as a single value, this can be broken down into seperate factors corresponding to the maximum contribution of any one gene (*maximum genetic contribution*) and the *maximum allelic contribution* within that gene. These two seperate factors are used in the derivation of the recessive model described below.

The maximum tolerated allele count (AC) was computed as the AC occurring at the upper bound of the one-tailed 95% confidence interval (95%CI AC) for the established maximum credible allele frequency, given the observed allele number (AN). Since the population is drawn without replacement, this would strictly be a hypergeometric distribution, but this can be modeled as binomial as the sample is much smaller than the population from which it is drawn. For ease of computation, we approximate this with a Poisson distribution. In R, this is implemented as `max_ac = qpois(quantile_limit,an*af)`, where `max_ac` is the 95%CI AC, `quantile_limit` is 0.95 (for a one-sided 95%CI), `an` is the observed allele number, and `af` is the maximum credible population allele frequency.

Defining maximum credible AF for recessive diseases

For a biallelic condition caused by only a single gene, the prevalence can be determined from the combined frequency of all possible pairs of alleles in that gene:

$$prevalence = \sum_{i,j} (AF_i \times AF_j \times penetrance_{i,j})$$

where $penetrance_{i,j}$ is the penetrance of two alleles i and j with frequencies AF_i and AF_j and i and j may be the same allele. This is generalisable in that all variants may be included in the summation, as the $penetrance_{i,j}$ will be 0 for benign variants, and those variants will not contribute to disease.

For a condition caused by multiple genes, the above equation must then be summed across n contributing genes:

$$prevalence = \sum_{1}^{n} \sum_{i,j} (AF_i \times AF_j \times penetrance_{i,j})$$

where $penetrance_{i,j}$ is the penetrance of i and j.

Since the penetrance cannot be separately estimated for each combination of alleles (though is typically high for recessive conditions), we simplify by estimating penetrance as constant for the condition, and calculating only for pathogenic alleles, reducing the equation to:

$$prevalence = penetrance \times \sum_{1}^{n} \sum_{i,j} (AF_i \times AF_j)$$

where $penetrance$ is the penetrance for any pair of pathogenic alleles and i and j are both pathogenic alleles.

Since we are now treating penetrance as constant for each gene this simplifies further: for a given gene containing m pathogenic alleles, the frequency of individuals who are homozygous or compound heterozygous is given by the square of the combined allele frequencies of all contributing alleles.

$$\sum_{i,j} (AF_i \times AF_j) = (\sum_{i=1}^{m} A\,F_i)^2$$

If we know the AF of a single variant i, and the contribution that variant makes to disease, then the combined allele frequencies of all contributing alleles ($\sum_{i=1}^{m} A\,F_i$) could in turn be represented as $\frac{AF_i}{allelicContribution_i}$ where $allelicContribution_i$ is the proportion of disease that is attributable to variant i.

Substituting this into our equation for prevalence yields:

$$prevalence = penetrance \times \sum_{1}^{n} (\frac{AF_i}{allelicContribution_i})^2$$

For a specific variant in a specific gene, with known allelic and genetic contribution (i.e. the proportion of cases attributable to the gene that contains variant i), prevalence can be expressed as:

$$prevalence = penetrance \times (\frac{1}{geneticContribution_i}) \times (\frac{AF_i}{allelicContribution_i})^2$$

Finally, we can rearrange this to give an upper bound for the maximum credible AF of an individual causative variant:

$$max\ credible\ AF = \sqrt{(prevalence)} \times maximum\ allelic\ contribution \times \sqrt{(maximum\ genetic\ contribution)} \times 1/\sqrt{(penetrance)}$$

where $maximum\ genetic\ contribution$ is the maximum proportion of cases attributatble to any one gene and $maximum\ allelic\ contribution$ is the maximum proportion of cases attributable to any one variant within that gene.

Curation of a high frequency PCD variant

NME8 NM_016616.4:c.271-27C>T which is reported as Pathogenic in ClinVar is found in 2306/120984 ExAC individuals. This variant was initially reported as pathogenic on the basis of two compound heterozygous cases when specifically searching for NME8 variants in a set of patients, and was found in 2/196 control chromosomes[1]. We further note that *NME8* has not otherwise been associated with PCD and shows no evidence of missense nor loss-of-function constraint and that this splice variant affects a non-canonical transcript. During our curation exercise we found that this variant meets none of the ACMG criteria for assertions of pathogenicity, and therefore we reclassified it as a VUS.

Dealing with penetrance

It is often difficult to obtain accurate penetrance information for reported variants, and it is also difficult to know what degree of penetrance to expect or assume in newly discovered pathogenic variants. In this work we uniformly apply a value of 50% penetrance for inherited cardiac conditions (i.e. assuming variant penetrance is no lower than 50%) equivalent to that reported for our HCM example variant, and the lowest we found reported for any of our examples or reported accross our studied disorders. We recognise several other approaches that can be used to deal with the issues of penetrance, these include: setting a penetrance level equivalent to the minimum that is 'clinically actionable' for a disorder; lowering the penetrance if reduced penetrance is expected in a family; or using a two tiered approach, initially searching for a high-moderate penetrance variant but allowing for a lower-penetrance variant in a second pass. We believe that the ease of re-calculating our "maximum credible population allele frequency" lends itself to any of these approaches. We provide an online calculator to facilitate the exploration of these parameters (http://cardiodb.org/alleleFrequencyApp). If there are large case and control populations for a disease and the diease prevalence is known, we can use these to estimate penetrance[2].

Treatment of singletons and other populations

It is worth considering whether a single observation in a reference sample should ever be treated as incompatible with disease. Using the approach outlined above, it can be inferred that an ExAC AC=1 would be considered incompatible with a true population allele frequency $<2.9\text{x}10^{-6}$ (with 95% confidence). For a penetrant disease with a prevalence of 1:1,000,000, the probability of observing a specific causative allele in ExAC is <0.01, even if the disease is genetically homogeneous with just one causative variant. In practice however, we feel that there are few, if any, diseases that are extremely rare yet have sufficiently well-characterized genetic architecture to discard singleton variants from a reference sample. Therefore, for singletons (variants observed exactly once in ExAC), we set the filtering allele frequency to zero.

We also note that occasionally a variant is seen in individuals falling under the Finnish or "Other" population categories in ExAC, and is a singleton or absent in all five continental populations. For these variants, the filtering allele frequency is set to zero. Because the Finnish are a bottlenecked population, disease-causing alleles may reach frequencies that would be impossible in large outbred populations. Similarly, because we have not assigned ancestry for the "Other" individuals, it is difficult to assess the population frequency of variants seen only in this set of individuals. Users are left to judge whether variants that would

not be filtered on the basis of frequency in the five continental populations, but that are sufficiently frequent in Finnish or "Other" populations, should be removed from consideration according to the specific circumstances.

Description of the filtering allele frequency

We define the "filtering allele frequency" for a variant, or `af_filter`, as the highest true population allele frequency for which the upper bound of the 95% confidence interval of allele count under a Poisson distribution is still less than the variant's observed allele count in the reference sample. It functions as equivalent to a lower bound estimate for the true allele frequency of an observed variant: if the filtering allele frequency of a variant is at or above the maximum credible allele frequency for a disease, then the variant is considered too common to be causative of the disease.

Consider, for example, a variant with an observed AC=3 and AN=100,000. If a user's maximum credible allele frequency for their disease is 1 in 100,000, then this variant should be kept in consideration as potentially pathogenic, because the upper bound of the Poisson 95%CI is AC=3. On the other hand, if the user's credible tolerated allele frequency is 1 in 200,000 then this variant should be filtered out, as the 95%CI upper bound is only AC=2. We define `af_filter` as the highest AF value for which a variant should be filtered out.

In the example, the highest allele frequency that gives a 95%CI AC of 2 when AN=100,000 is approximately 8.17e-6. Instead of solving exactly for such values, which would require solving the inverse cumulative distribution function of the Poisson distribution, we derive a numerical approximation in two steps:

1. For each variant in consideration, we use R's `uniroot` function to find an AF value (though not necessarily the highest AF value) for which the 95%CI AC is one less than the observed AC.
2. We then loop, incrementing by units of millionths, and return the highest AF value that still gives a 95%CI AC less than the observed AC.

In order to pre-compute `af_filter` values for all of ExAC (verson 0.3.1), we apply this procedure to the AC and AN values for each of the five major continental populations in ExAC, and take the highest result from any population. Usually, this is from the population with the highest nominal allele frequency. However, because the tightness of a 95% confidence interval in the Poisson distribution depends upon sample size, the stringency of the filter depends upon the allele number (AN). The stringency of the filter therefore varies appropriately according the the size of the sub-population in which the variant is observed, and sequencing coverage at that site, and `af_filter` is occasionally derived from a population other than the one with the highest nominal allele frequency.

For this analysis, we used adjusted AC and AN, meaning variant calls with GQ≥20 and DP≥10.

Analysis tools

R was used for all analyses, as well as to compile the figures, manuscript and supplementary material, making use of the following packages: knitr/rmarkdown (https://github.com/yihui/knitr), knitcitations (http://www.carlboettiger.info/2012/05/30/knitcitations.html), knitauthors (https://github.com/jamesware/knitauthors), dplyr (https://github.com/hadley/dplyr), ggplot2 (http://ggplot2.org/), packrat (https://rstudio.github.io/packrat/).

SUPPLEMENTARY TABLE S1

Variants previously reported as causative of HCM either in ClinVar, or in a clinical series of 6179 HCM cases, but that were observed in ExAC above the maximum tolerated allele count for HCM (AC>9 globally) were manually curated according to the ACMG guidelines for interpretation and classification on sequence variants. For 5/43 variants, ClinVar contained the only identifiable disease-associated variant record, while 38/43 had additional information in the literature. Curated variant interpretations are deposited in ClinVar under the submission name "HCM_ExAC_frequency_review_2016". *These variants were previously categorised as Likely Pathogenic in the LMM and Oxford HCM cohorts.

measureset_id	symbol	hgvs_c	ExACCount	Class	Notes
43475*	MYL2	c.401A>C	19	VUS	8/8 missense prediction algorithms predict deleterious. Identified in 2 individuals with a second 'likely pathogenic' MYL2 variant (ClinVar SCV000060059.4). An in vitro functional study suggests that this variant may reduce cardiac muscle contraction efficiency (Burghardt 2013). Second hit causing more severe phenotype suggested. [BS1 + PP3]
43121*	MYL3	c.170C>A	14	Likely Benign	8/8 missense algorithms predict deleterious. Mutations at the same residue have been reported for HCM (Lee 2001). Indentified along with with pathogenic and likely pathogenic variants (ClinVar SCV000059671.4, SCV000207109.1). 2 affected siblings reported both with variant as homozygote (no support for recessive MYL3 inheritance). [BS1 + BP5 + PP3]
180694	MYOM1	c.1900+3A>C	466	Likely Benign	Originally identified as a homozygote in 3 yr old (ClinVar SCV000195854.1). No other data to support. 7 homozygotes in ExAC. Splicing tools predict no effect. [BS1 + BP4]
41926	CRYAB	c.460G>A	93	Likely Benign	Identified in a HCM case with an alternative variant sufficient to cause disease (ClinVar SCV000060874.4). Previous pathogenic assertion in ClinVar is from 'literature only' for DCM (no segregation, only 200 controls used for reference). All others say 'VUS' [BS1 + BP5]
12411	TNNT2	c.853C>T	40	VUS	Reported in >10 individuals with HCM and segregated in 5 affected individuals from multiple families (Watkins 1995, Elliott 1999, Garcia-Castro 2003, Torricelli 2003, Van Driest 2003, Theopistou 2004, Ingles 2005, Zeller 2006, Garcia-Castro 2009, Kaski 2009, Gimeno 2009, Millat 2010). Up to half of individuals carried a second variant (ClinVar SCV000060277.4). Assuming a prevalence of 1/500 and a max allelic contribution of 2%, for this variant to have a role in disease we would calculate a maximum penetrance of 6.2%. Residue not evolutionarily conserved. A milder affect with late age of onset has been suggested. In vitro

					studies suggest may have an affect on muscle contraction (Morimoto 1999, Yanaga 1999, Szczesna 2000, Hernandez 2005) however these might not represent acurately protein function. [BS1]
177700	MYBPC3	c.2441_2443delAGA	39	VUS	In-frame deletion. Experimental studies have shown that this deletion does not lead to decreased MYBPC3 stability in vitro (Bahrudin 2008). Has been identified in 4 adult unaffected relatives (ClinVar SCV000203960.3). [BS1 + PM4]
14064	MYL2	c.37G>A	37	Benign	ClinVar 'Pathogenic' assertion from literature only (ClinVar SCV000035365.1). Non-segregation: absent in an affected family member and found in a healthy 56-year-old male (Ball 2012). Found in Ashkenazi Jewish individuals. Reported to lead to increased Ca2+ affinity (Szczesna 2001). [BS1 + BS4]
161310	MYBPC3	c.961G>A	37	Likely Benign	Evidence based on presence in HCM individuals (1 with a MYH7 variant (Maron 2012)) and absence in ESP and nearby residues reported as pathogenic (Ser311Leu and Arg326Gln). No segregation or functional data. Reported multiple times in ClinVar with other variants that could explain phenotype (SCV000208258.4, SCV000261724.2). [BS1 + BP5]
177902	MYBPC3	c.1000G>A	36	VUS	Very high freqeuncy in East Asians (0.45% in ExAC). Reduced stability of MYBPC3, higher Ca2+ transients and action potential durations in HL-1 cells and rat cardiac myocytes (Bahrudin 2011). [BS1]
161305	MYBPC3	c.13G>C	36	Likely Benign	Reported in two families; found in one proband and absent in their unaffected mother, and found in another proband and absent in unaffected sibling. 3 individuals with variant also carry a second pathogenic mutation in the same gene (ClinVar SCV000198995.3). Amino acid not conserved in mammals. [BS1 + BP5]
191577	ANKRD1	c.368C>T	28	Likely Benign	8/8 missense algorithms predict benign. Mutant protein correctly incorporates into the sarcomere (Crocini 2013). Amino acid change is not conserved. [BS1 + BP4]
14149	MYH6	c.3195G>C	28	VUS	6/7 missense algorithms predict deleterious. Originally identified in one proband (died of congestive heart failure at 45 yrs) and absent in 2 unnafected offspring and 150 unrelated controls (Carniel 2005). Amino acid change seen in birds. [BS1 + PP3]
12445	TNNC1	c.435C>A	28	VUS	Functional studies using mutant porcine cardiac skinned fibers showed significantly increased force development and force recovery compared to

					wildtype (Landstrom 2008) with reduced Ca2+ binding [only in vitro studies]. No segregation data. Has also been reported in a patient with idiopathic DCM who also harbored a missense variant in MYBPC3 gene (Pinto 2011) [BS1]
42900	MYH7	c.2360G>A	27	Likely Benign	11 reports in total (7 literature, 4 LMM in ClinVar (SCV000059440.4)), majority South Asian decent - very high South Asian frequency (0.1%). 3 probands had another variant sufficient to cause disease. Only moderately conserved amino acid. Possible non-segregation (absent in one proband's father who died of sudden cardiac death, and therefore thought to be possibly affected; ClinVar SCV000059440.4). [BS1 + BP5]
43911	ACTN2	c.1484C>T	26	Benign	4 individuals in the literature (Chiu 2010, Theis 2006) and 2 reported on ClinVar (SCV000060535.4). Non segregation with disease also reported (2 affecteds; ClinVar SCV000060535.4). No supporting segregation data. [BS1 + BS4]
42536	MYBPC3	c.1468G>A	25	Likely Benign	7/7 missense algorithms predict deleterious. Identified in individuals with HCM, DCM and LVNC. Conserved residue. Multiple reports of co-occurance with an alternative variant that is sufficient to cause disease (ClinVar SCV000059050.4). [BS1 + BP5 + PP3]
31766	MYL2	c.141C>A	22	Likely Benign	7/8 missense algorithms predict benign. Co-occurance with likely pathogenic (tested) MYH7:c.5134C>T mutation (segregation data) in literature (Andersen 2001, Hougs 2005). Other reports of a second variant (ClinVar SCV000060036.4). In vitro studies show reduced force development and power output (Szczesna-Cordary 2004, Abraham 2009, Greenberg 2009, Greenberg 2010). [BS1 + BP5 + BP4]
44710	TCAP	c.458G>A	20	Likely Benign	7/8 missense algorithms predict benign. Only pathogenic in ClinVar by 'literature only'. Identified in siblings but no strong segregation data (Hayashi 2004). Functional studies in yeast show the variant leads to an increase in the ability of TCAP to bind with titin and CS-1 (Hayashi 2004)- in vitro assay may not accurately represent biological function. Amino acid change is not conserved - 7 mammals carry the variant amino acid. [BS1 + BP4]
36601	MYBPC3	c.1321G>A	18	Likely Benign	Multiple reports of co-occurence with alternative pathogenic variants (ClinVar SCV000059030.4 and SCV000208011.4). Computational modelling data suggest mutation may result in a confirmational change in the protein and a change in surface interactions (Gajendrarao 2015). [BS1 + BP5]
42775	MYBPC3	c.624G>C	18	VUS	Reports of possible non-segregation (ClinVar SCV000284248.1). [BS1]

14111	MYH7	c.2183C>T	18	Likely Benign	Pathogenic in ClinVar from 'literature only' - VUS by 3 other submitters. Identified originally in cis with another MYH7 mutation in 2 siblings with HCM (Blair 2001) - other variant (c.1816G>A) is absent in ExAC and has been shown to segregate in multiple families (24 literature reports) [BS1 + BP2]
4243	MYLK2	c.260C>T	16	VUS	Pathogenic in ClinVar from 'literature only', no other submissions. Found as a compound het in a 13yr old and with an MYH7 variant (also in mildy affected parents; Davis (2001). Other compound het variant is also common. [BS1]
12197	VCL	c.2923C>T	16	VUS	Pathogenic in ClinVar from 'literature only' .Reduced levels of vinculin in the patient with this variant, but other studies found that this was a general feature of HCM and not related to specific genotypes (Vasile 2006). [BS1]
14122	MYH7	c.1322C>T	16	VUS	Pathogenic in ClinVar from 'literature only', in Laing distal myopathy. No change in mRNA levels. Amino acid is not conserved in mammals. [BS1]
30143	MYBPC3	c.2618C>A	13	VUS	Identified as a compound het and in homozygous state (consaguinous family; Nanni 2003, Ingles 2005). No segregation data in HCM. 1 homozygote seen in ExAC. [BS1]
31780	MYL3	c.170C>G	11	VUS	7/8 missense algorithms predict damaging. Functional study showing lowercomplex affinity not significant for this variant after multiple testing (Lossie 2012). Segregation in 5 affected members of 2 families (ClinVar SCV000199362.3). All reports in East Asians (highest ExAC frequency at 0.05%). [BS1 + PP3 + PP1]
45533	LDB3	c.1823C>T	11	VUS	Only HCM report on ClinVar is labeled as 'literature only'. Associated reference contains no additional evidence for pathogenicity ie. no segregation or functional data. [BS1]
42679	MYBPC3	c.3049G>A	10	Likely Benign	Not conserved amino acid - 7/7 missense algorithms predict benign. No functional or segregation data. [BS1 + BP4]
9380	SCN5A	c.4534C>T	9	VUS	8/8 missense algorithms predict damaging. Reported in association with Brugada syndrome (Rook 1999, Desch�nes 2000, Meregalli 2009, Crotti 2012). In vitro functional studies show a potential impact (Rook 1999, Desch�nes 2000), however these may not be representative of in vivo protein function. [BS1 + PP3]
6055	KCNE2	c.79C>T	9	VUS	7/8 missense algorithms predict damaging. Identified in 2 Chinese families for Atrial Fibrillation (Yang 2004). Only parent and child tested for mutation (50%

					chance of segregation). An in vitro study on rat cardiomyocytes saw supression of L-type Calcium current (Liu 2014). [BS1 + PP3]
164023	MYBPC3	c.3763G>A	9	VUS	1/5 individuals with variant also had a likely pathogenic variant in another gene (ClinVar SCV000198796.2). No other data available. [BS1]
161309	MYBPC3	c.2269G>A	8	VUS	Conservative amino acid change. No functional or segregation data found. [BS1]
42664	MYBPC3	c.2873C>T	7	Likely Benign	2 documented individuals also carry a MYBPC3 variant sufficient to explain disease (ClinVar SCV000059185.4). Amino acid is not well conserved 7/7 missense algorithms predict benign. [BS1 + BP4 + BP2]
48097	LMNA	c.976T>A	7	Likely Benign	Position not conserved 7/8 missense algorithms predict benign, simulation studies suggest no effect on coil B dimerisation. [BS1 + BP4]
42566	MYBPC3	c.1786G>A	7	VUS	6/7 missense algorithms predict damaging. No clear segregation data (one suggestion of non-segregation (ClinVar SCV000259413.1)). [BS1 + PP3]
36613	MYBPC3	c.529C>T	6	Likely Benign	Different missense at the same position is common. Identified with another pathogenic MYBPC3 variant (ClinVar SCV000198969.3). Otherwise found in DCM. Position not well conserved. [BS1 + BP5]
180968	MYBPC3	c.2381C>T	6	VUS	No evidence towards a pathogenic classification found. [BS1]
42756	MYBPC3	c.461T>C	5	Likely Benign	Position not conserved, 6/7 missense algorithms predict benign.Identified in a child with a further homozygoes MYBPC3 variant (ClinVar SCV000059282.4). [BS1 + BP4]
177629	MYH7	c.5326A>G	4	Likely Benign	Large majority of identified cases are of Asian ancestry. 2 individuals at GeneDx have an alternative pathogenic variant (ClinVar SCV000208629.3). Only segregation data is in 2 affected siblings and not in the unaffected sibling (Blair 2002). [BS1 + BP5]
191705	MYBPC3	c.640G>A	4	VUS	No evidence towards a pathogenic classification found. [BS1]
14147	MYH6	c.2384G>A	4	VUS	Identified in a 75 year old patient and not found in 170 controls (Niimura 2002). No other data. [BS1]
161313	MYBPC3	c.223G>A	3	VUS	No evidence towards a pathogenic classification found. [BS1]
42706	MYBPC3	c.3330+5G>C	2	Pathogenic	Prevalence in affecteds statistically increased over controls (Walsh et al. Genet Med. 2016). Shown to produce a truncated protein product and to segregate with disease (Watkins 1995). [BS1 + PVS1 + PS4 + PP1]

SUPPLEMENTAL REFERENCES

1. Duriez, B. *et al.* A common variant in combination with a nonsense mutation in a member of the thioredoxin family causes primary ciliary dyskinesia. *Proceedings of the National Academy of Sciences* **104,** 3336–3341 (2007).

2. Minikel, E. V. *et al.* Quantifying prion disease penetrance using large population control cohorts. *Science Translational Medicine* **8,** 322ra9–322ra9 (2016).
